# With Corona Outbreak: Nature Started Hitting the Reset Button Globally

**DOI:** 10.3389/fpubh.2020.569353

**Published:** 2020-09-24

**Authors:** Ashwani Kumar, Muneer Ahmad Malla, Anamika Dubey

**Affiliations:** ^1^Metagenomics and Secretomics Research Laboratory, Department of Botany, Dr. Harisingh Gour University (A Central University), Sagar, India; ^2^Department of Zoology, Dr. Harisingh Gour University (A Central University), Sagar, India

**Keywords:** SARS virus, COVID-19, environmental pollution, pandemic, respiratory diseases

## Abstract

Considering the potential threat and the contagious nature of the Covid-19 pandemic, lockdowns have been implemented worldwide to stop the spread of this novel virus. The coronavirus pandemic has hit the world severely, representing the most severe threat to human health in more than a century. The environment from local to global scales has witnessed apparent positive and negative impacts. Global lockdowns have drastically altered the patterns of energy demand and have caused an economic downturn but at the same time, have provided an upside-cleaner global environment. Such immense unintended advantages offer opportunities for unprecedented insights into the dynamics of our natural and built environments that can lead to viable paths for the conservation and perpetuation of the recovered environments and through sensible policies and practices that can help to create new recovery pathways. Knowledge gained from the studies suggests that a substantial relationship exists between the contingency measures and environmental health. Here in this review, the authors discussed the impact of coronavirus pandemic on human life, healthcare organizations, and the environment. The parallels between the Covid-19 and other diseases are mentioned. Finally, the impact of Covid-19 on society and the global environment has also been highlighted.

## Introduction

**Figure 1:**
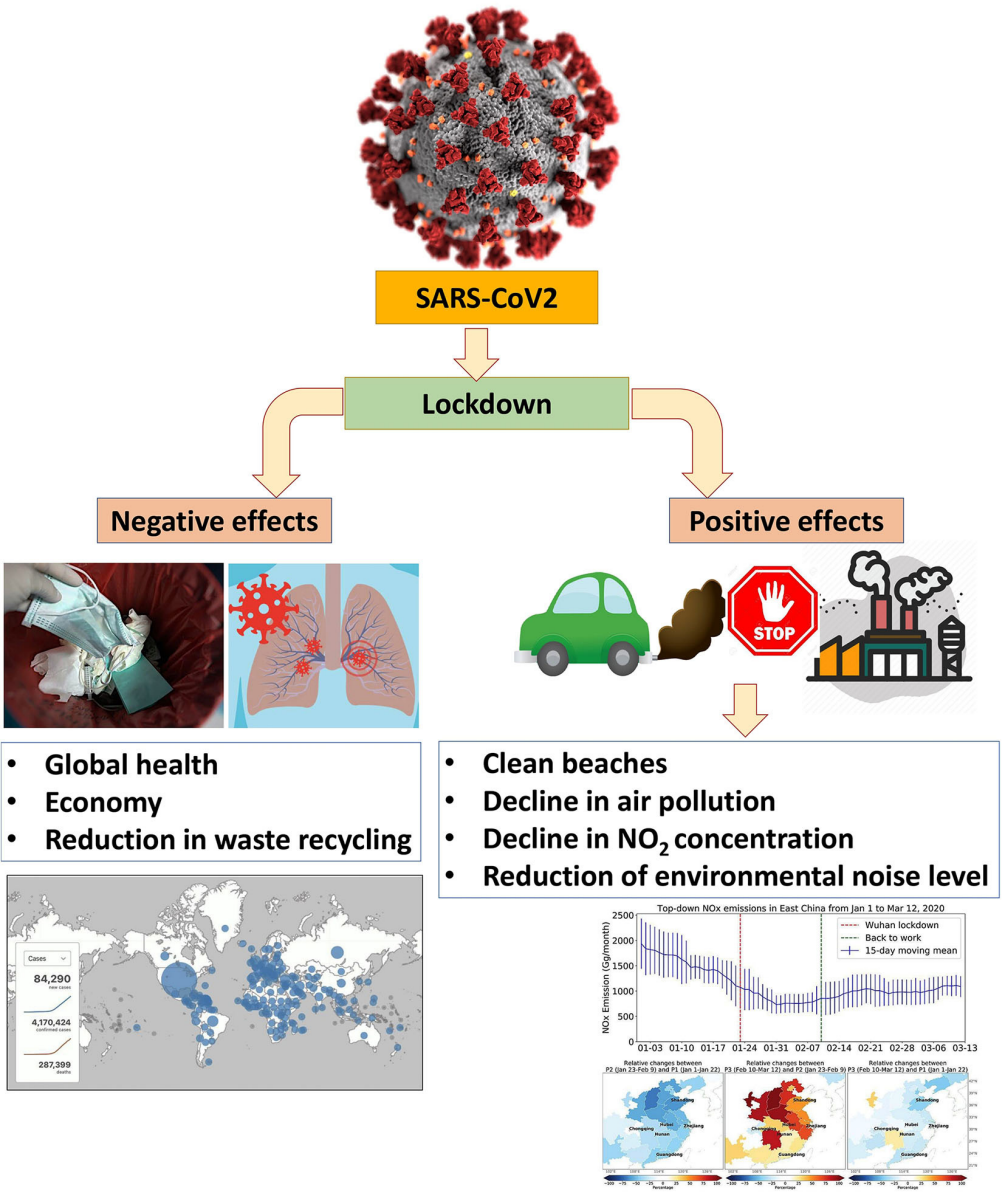
The positive and negative effects of Covid-19.

**Figure 2:**
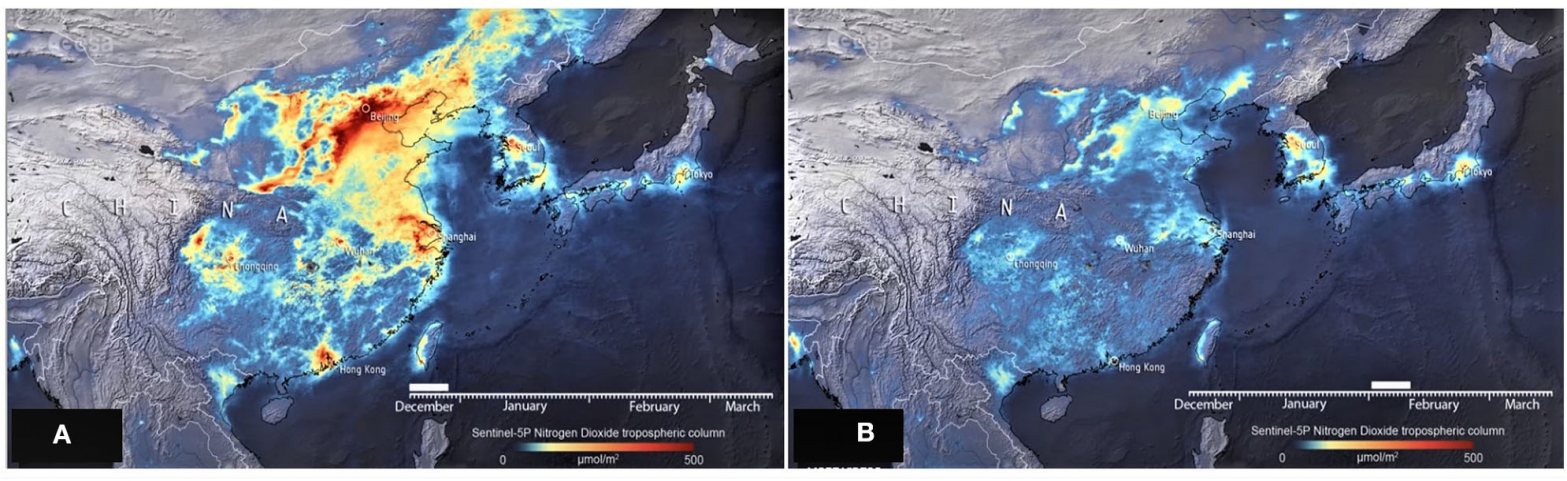
NO_2_ drops down in the coronavirus epicenter Wuhan, *Hubei Province*-China. **(A)** In December 2019, **(B)** February 2020 (4).

Coronaviruses belong to the group of viruses with subfamily *Coronavirinae* within *Coronaviridae* family and are deemed as possible agents of respiratory diseases with symptoms such as flu, fever, runny nose, cough, breathing difficulty, pneumonia, and lung infection (1). In December 2019, a novel coronavirus disease (Covid-19) originated, in Wuhan, Hubei province, China, and soon sprouted across the globe (2). By February 2020, the daily number of Covid-19 cases outside China had increased drastically, with Italy, USA, Spain, Germany, South Korea, Japan, and Iran being the new major epicenters. Based on the alarming levels of spread and severity, on 11 March 2020, the world health organization (WHO) characterized the Covid-19 situation as a pandemic, and by the end of March 2020, Europe emerged as the new hotspot and was declared as the world's major epicenter (3). As of 14 July, the Covid-19 disease has spread to more than 200 countries and Union Territories (**Figure 2**), with over 13,177,855 confirmed cases and over 574,793 confirmed deaths worldwide (2). As this global pandemic hits more than 200 countries, the virus besides taking a huge toll on public health has completely hijacked the rhythm of our daily lives, hit the global economy, and forced the countries to shut their borders (2, 5). Data released by European Space Agency (ESA) and National Aeronautics and Space Administration (NASA) indicates that pollution in some of the epicenters of Covid-19 such as Wuhan, USA, Spain, and Italy has decreased by up to 30% (3). With the USA, Spain, Italy, UK, France, and Germany, among the worst-hit countries in terms of infections and deaths, India is also facing the heat, and the figures too are no less devastating. Environmental pollution has its roots in industrialization and urbanization, which are the main sources of the huge release of greenhouse gases (6). The majority of the studies conducted in the recent past have focused more upon fighting against this deadly virus but very few are focused on the indirect beneficial effect of this pandemic on the environment (3, 6, 7). Climate experts predicted that the emission of greenhouse gases (GHGs) could fall by large amounts (nearly 8%) since World War II (8). This reduction in the level of GHGs is a consequence of lockdown and social distancing policies adopted by governments in different countries to combat the coronavirus spread. These lockdown measures severely affect the country's main commercial activities (9). As a result, industrial facilities and power plants stopped their production and uses of vehicles decreased considerably. This led to an intense decline in the concentrations of particulate matter and nitrogen dioxide (NO_2_) in China and the reduction of air pollution in Europe. Therefore, in this review, we discussed both direct or indirect positive and negative effects of the Covid-19 pandemic on the environment and human health (Figure 1).

## Corona Pandemic and Human Life: A Growing Misery

The pandemic turn's messier and everyday life suffers, as the world remains under lockdown (9). As mentioned above, till this writing, the number of Covid-19 cases worldwide was 12,977,429, with over 570,259 fatalities (https://coronavirus.jhu.edu/) and over 2.5 billion people around the world under lockdown and stay-at-home orders (2). In the present era, in which we live today, the development is at its peak, with quality and sophistication of the technologies and medicines at their best. Moreover, we are also witnessing a rapid and efficient collaboration and communication of nations at global levels, yet this Covid-19 pandemic is sweeping throughout the globe with high mortality, grounding most of the global population, bringing the healthcare systems of the world's developed countries to a breaking point, and shattered our views of normality and peaceful life (10–12). It is quite enough to say, that the combination of a deadly and rapidly propagating virus and the weak health infrastructures, less information about this virus, and unavailability of vaccine and proper medication, have created the perfect storm and the people are in a panic. Covid-19 pandemic has been an urgent wake-up call for many developed and rich countries, in terms of their failure to stop this pandemic and save the lives of their citizens, as a result, has brought forth their fragile healthcare systems (13). Lack of effective testing, understaffed hospitals, precise and science-oriented guidance and directions, crumbling healthcare systems, and early planning to procure necessary medical equipment [like personal protective equipment (PPE)], despite increasing evidences that the world is facing a global health emergency has been shocking in most of the developed countries especially in the USA, EU, and KU (14). These countries, being superior in terms of their unions, quality and strength of medical experts and researchers, economic strength, and better healthcare systems, failed enormously in dealing with this pandemic (15). This have unfortunately placed the weak and marginalized section of the population at greater risk.

## Parallels Between Covid-19 Pandemic and Other Global Crisis

The present Anthropocene is witnessing an upliftment of global crises such as climate destabilization, population explosion, conflicts, increasing levels of inequality among the people, economic uncertainty, mounting public health threats, and most recently the Covid-19 pandemic (3). All these global crises are slowly tipping the balance, questioning the global economy, financial markets, and public health thereby forcing the society to rethink the next steps. There are certain degrees of parallels that can be drawn between the ongoing Covid-19 pandemic and some other contemporary crises the world is currently facing. All of these necessitate long-term thinking and global response guided by science to address these crises. In this view, the Covid-19 pandemic may provide an opportunity to the scientific community and the political system for a much deeper understanding of the global crises and could help us to tackle the biggest threat of the century, the climate crisis. According to Wyns, a member of the climate change panel at the WHO, the world is witnessing overwhelming consequences of the under-prepared health systems because of these shocks, with most of these having a clear climate change signature. Almost all the health shocks have one thing in common; they hit the poor, vulnerable, and marginalized the hardest (2). At least 50% of the global population does not have the most basic health services, therefore when a disaster hits, global inequality is sustained, and compensated with the lives of the weak and marginalized sectors of the society (11, 13). The same stands true for climate change, e.g., the burning of fossil fuels besides adding to air pollution disproportionately impacts the health of poor and weaker people. Secondly, the Covid-19 pandemic like other global crises has crushed the entire global development with records of economic recession and financial meltdowns. This has put the fate of not only the poor but also the rich countries under such a threat that the world has never seen. With international trade slowing down, commodity prices collapsing, the third-world countries that were already in misery are on the edge of full-blown sovereign debt crises (16).

## Measures to Slow Down the Coronavirus

With the world locked up in a deadly fight against the Covid-19 pandemic, countries across the globe are setting standard measures to slow the spread of this virus. Without enough test kits, the low-income countries are mainly relying on the healthcare workers to trace, quarantine, and self-isolate the people to slow down the rate of infections. In these countries, people only get tested if they develop symptoms (15, 16). Experts predict that the stakes are high enough if the present containment measures fail to slow the spread of this deadly viral outbreak. Some of the main measures taken by the governments to slow down the outbreak are contract tracing, travel restrictions, social distancing, temperature checks, widespread testing, and ban on gathering, closing of educational institutes, lockdowns, and self-quarantine (15).

## Covid-19 and the Global Climate Change

Irrespective of the obvious decline in CO_2_ emission and air pollution owing to the lockdowns which although temporarily, but may contribute to mitigating climate change (17, 18), many parallels indeed do exist between the challenges in fighting this global climate change and pandemic. As discussed, the mandatory lockdowns have recorded up to a 5°C reduction in temperature than the prelockdown periods, indicating that the industrial sector is likely responsible for the energy footprints that can dramatically increase the temperature. Since, both climate change and pandemic are existential challenges that the human race is facing. Neither the coronavirus pandemic nor the climate sees the continental borders, as is evident from the current Covid-19, floods in midwest plains, bush fires in Australia, droughts in California, growing deserts in Central Asia, retreating glaciers in the Alps, and the melting polar ice caps, and the consequences of climate change will affect all of us (humans) in some form at some point and no one can escape these consequences (19). Therefore, there is a need to consider all these problems as “our problems” and its urgent time to think and act together. The catastrophic consequences of climate change are quite severe and can damage the environment and biodiversity. However, learning from the lessons of Covid-19, we must act now to avoid any further global catastrophe and be aware of the sinister threats that may arise gradually. Similarly, ignoring the ever-growing scientific evidence of both climate change and the Covid-19 pandemic cannot save us from the hazardous consequences (20, 21). Therefore, the need of the hour is to make decisions based on scientific evidence. Fighting any global disaster needs international collaboration so that scientists can work together and address the challenges. In the case of the present coronavirus pandemic, the global collaboration is impressive, similarly, the modeling and understanding of climate change issues are a global collaborative effort by the Intergovernmental Panel on Climate Change (IPPC) (17).

## Positive and Negative Impacts of Covid-19

Without a viable therapy and vaccine, the international community is on tenterhooks trying to limit the spread of coronavirus and reduce the mortality from the Covid-19 pandemic. The virus has quickly impacted the government and public health systems and forced the governments to declare a national and international public health emergency. Given the restrictions in public movement, closed borders, reduced public transport, halted non-essential services, and shelter-in-place orders, the planet is witnessing both the positive and negative effects of the Covid-19 pandemic (3).

## Positive Effects of Covid-19 on the Environment

### The Planet Earth: An Unlikely Beneficiary of Corona Pandemic

With the global lockdown in process, the Internet is abounded with articles and the social media outlets with pictures, showing the planet being the unlikely beneficiary of this Covid-19 pandemic (22). Nature seems to have hit the reset button, reclaiming the spaces to heal itself as the anthropogenic activities have slowed down. Amidst all the gloom and doom that the Covid-19 pandemic is giving, there seems to be a proverbial silver lining and some positive consequences as well (22). Some of these are mentioned below:

### Decrease in Air Pollution Level

**Figure 3:**
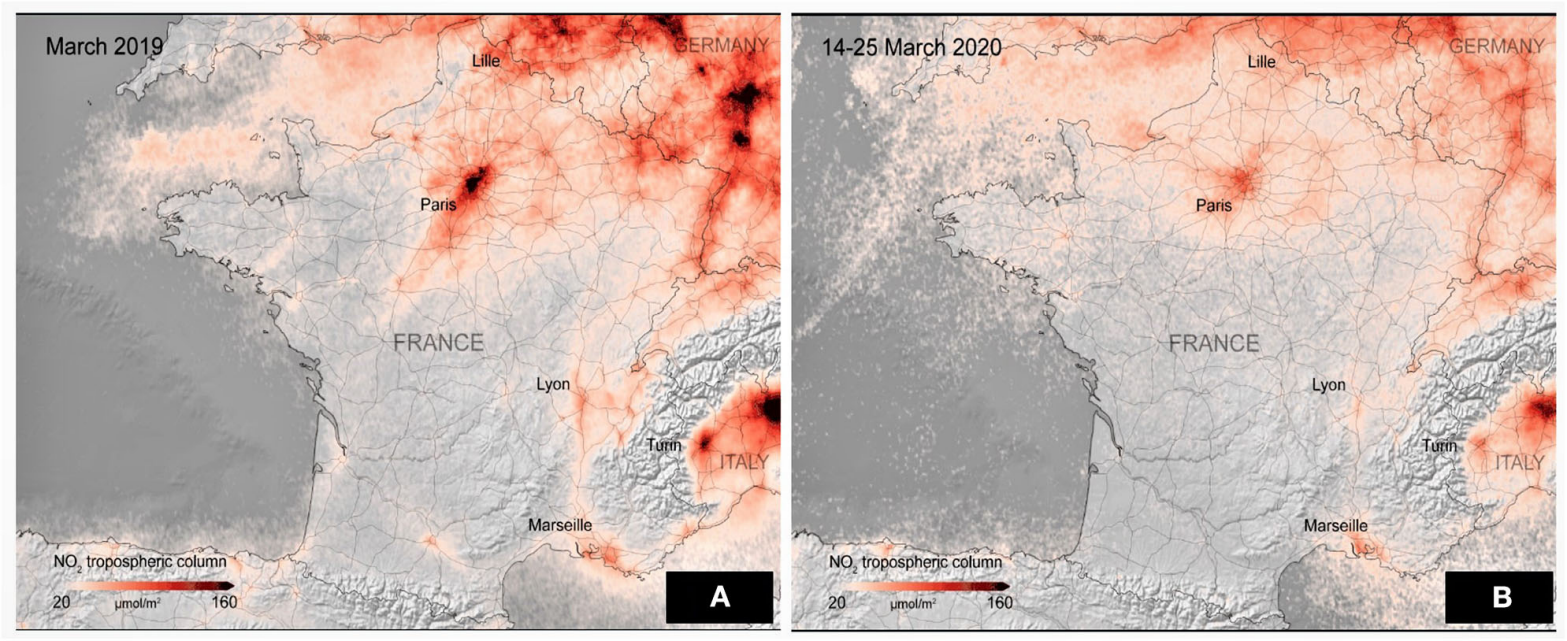
Satellite images from ESA showing a dramatic reduction in the amount of harmful greenhouse gas emissions in the atmosphere. Pollution drops down in European countries amid coronavirus quarantine **(A)** As on March 2019, and **(B)** March 2020 (26).

**Figure 4:**
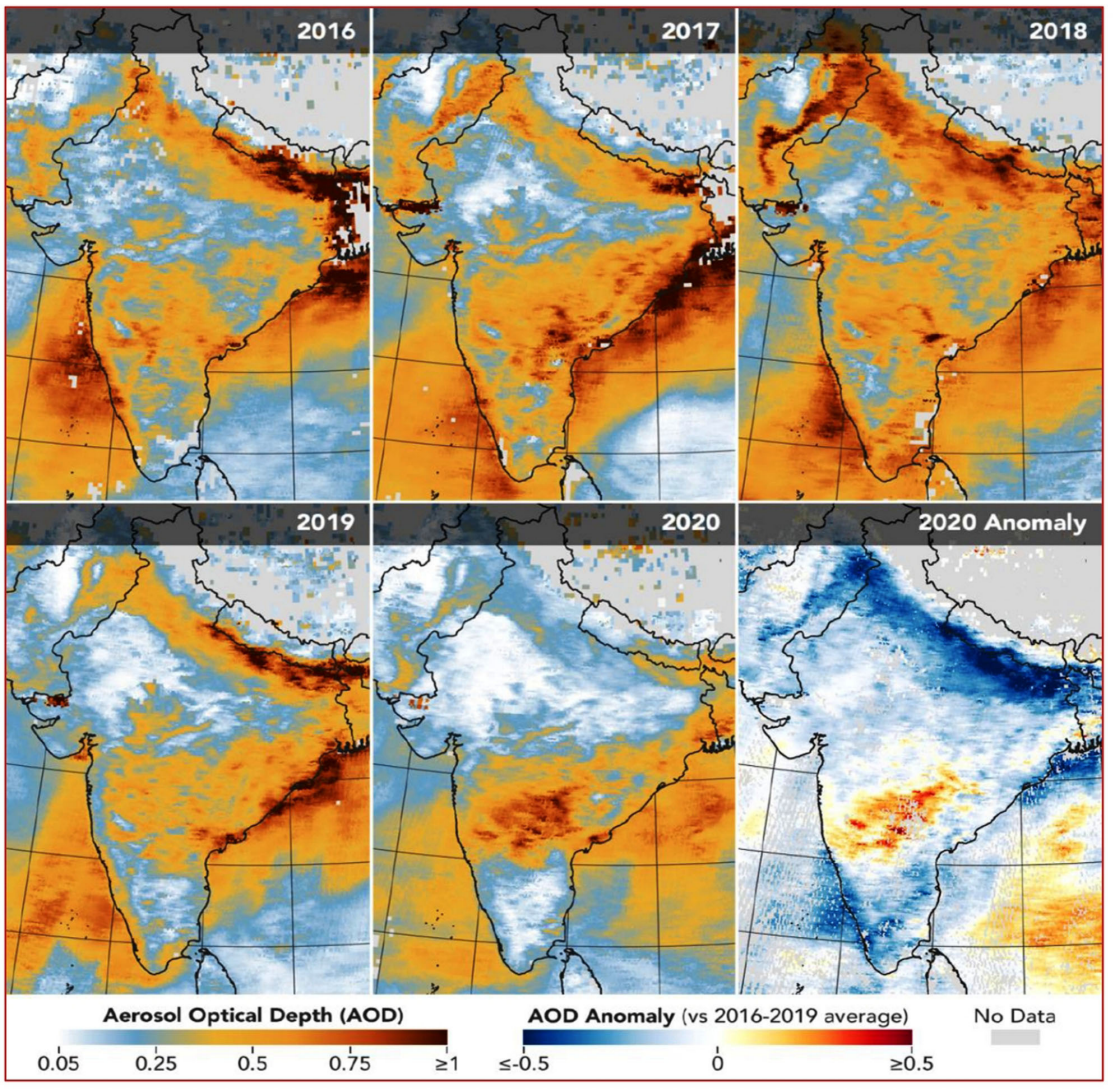
Pre- and post-lockdown level of airborne aerosols over India Source: NASA and ESA, (source, https://earthobservatory.nasa.gov/images/146596/airborne-particle-levels-plummet-in-northern-india, https://earthobservatory.nasa.gov/. accessed date: 13 May 2020).

Air comprises the immediate environment of human beings, which is vital for their survival. With 91% of the global population living in places where the air quality is poor, with Air Quality Index (AQI) exceeding the permissible limits (23), the possible health effects of the degraded air quality had the largest footprints attributable to the pervasive, pernicious, prolonged, and constant exposure to pollution. Although, the possible health effects of pollution in general and air pollution, in particular, are considered the tip of the iceberg, however, the consequences of the global air pollution are manifested in terms of the significant percentage of deaths worldwide each year (24). The Lancet commission reports on pollution and health suggest that pollution accounts for more than 16% of the global deaths, with air pollution alone contributing up to 8% of these deaths, which is three times more than the deaths due to tuberculosis, malaria, and AIDS and 15% more than warfares and other global violence (24, 25). Estimates suggest that more than 90% of the pollution-related deaths occur in developing countries, such as Asia and Africa. The United Nations (UN) General Assembly has already adopted 17 sustainable development goals (SDGs) related to climate change. The UN general assembly has adopted additional SDGs on clean air, clean water, good health, responsible production, and the industrialization of cities, marine, and terrestrial life (24, 25). In response to the current Covid-19 pandemic and with countries suspending transport and millions of people put in lockdown to flatten the curve, global air pollution has significantly come down, with carbon monoxide emission reduced by more than 50%. China was the first country to implement self-quarantine measures and strict traffic restrictions to control the expansion of Covid-19. This global ban on traffic mobility and lockdown greatly limited transportation emissions and declined industrial and residential heating. These actions were found to generate changes, as reported by NASA and ESA. The level of NO_2_ was reduced by 12.9 and 22.8 μg/m^3^ in China and Wuhan city in Hubei Province, respectively [(4), Figure 2]. Similarly, particulate matter (PM 2.5) was found to reduce by 1.4 μg/m^3^ in Wuhan but showed a significant dropdown (18.9 μg/m^3^) in the majority (more than 350) of the cities. Figure 3 clearly illustrates a sharp reduction in NO_2_ concentrations in other European countries such as Germany, Italy, Spain, and France [(26), Figure 3]. The dramatic increase in the air quality level across China during the quarantine period was also detected by the Copernicus Sentinel-5P satellite. Similarly, the data from the Copernicus Sentinel-5P satellite, using the nitrogen dioxide tropospheric column density, revealed a steep decline in air pollution, particularly in the NO_2_ emissions, over Italy, post-corona lockdown. Additionally, based on the reports of Copernicus Atmosphere Monitoring Service (CAMS), the European Union observed a significant drop in PM 2.5 (20–30% approx.) in February 2020 compared with the monthly average of 2019, 2018, and 2017 (27, 28) (Table 1). According to Fei Liu, an air-quality scientist at NASA's Goddard Space Flight Center, such dramatic dropoff in the air pollution was seen for the first time from January 2020. China also witnessed a significant dropdown (36%) in coal-fired power from 3 February to 1 March 2020 (28). Coronavirus has cut emissions faster than years of climate negotiations. In India, like in the rest of the world, with strict lockdown in place and with a lesser number of people venturing, the country has seen a drastic fall in pollution levels. The AQI lowered from 500 to 600 in winters, to as low as 50 in April (Figure 4) (https://www.aqi.in/). In China alone, all the interventions to contain the severe acute respiratory syndrome coronavirus (SARS-CoV)-2 outbreak led to air-quality improvements with prominent health benefits that outnumbered the confirmed Covid-19 deaths (28).

**Table 1:** Reduction in particulate matter (PM 2.5).

**Countries**	**Average PM 2.5 during lockdown 2020 (μg/m3)**	**Reduction compared with 2019 (%)**	**Reduction compared with prior 4-year average (%)**
Los Angeles, USA	5.5	−31	−51
UK	16.2	−9	+6
China	35.1	−44	−50
Italy	16.7	+30	ND
Spain	6.4	−11	+2
New York, US	4.4	−25	−29
Brazil	10.1	−32	−26*
South Korea	24.1	−54	−32
India	32.8	−60	−55

*ND, no data*.

* *data is compared on the basis of a 3-year average rather than a 4-year average. Source, https://www.iqair.com/world-air-quality-ranking*.

As mentioned, the locking down of cities has significantly improved the environmental quality with a sharp drop in air pollution levels across several countries (2, 6, 29–33). Whereas, before and after lockdown comparison is problematic because it lacks proper counter facts. However, despite a return to normalcy, and easing of restrictions, there is evidence to suggest that the air quality has considerably improved after the lockdown (31). Data analysis from different countries shows low NO_2_ pollution levels (30% below the normal level at the end of June) despite traffic and commercial operations being back to normal. Moreover, studies suggest that more developed countries could be more substantially influenced by lockdown, as industrial activities remain largely suspended (16). Similarly, the lockdown effects are larger in rich and cold areas and cities with more traffic volumes experience a more substantial reduction in air pollution because richer countries have higher electricity demands and colder areas have higher coal demands, respectively (29–33). Data like these hint that mother earth is an unintended beneficiary of the Covid-19 pandemic.

### Environmental Noise Pollution Reduction

Environmental noise pollution is well-defined as an undesirable sound generated by various anthropogenic, transport, industrial, and commercial activities and is the major source of discomfort for the environment and human health (3, 29). Prolonged exposure to noise pollution has been shown to cause a range of health problems such as stress, tinnitus, cognitive impairment, cardiovascular disease, hearing loss, lack of sleep, fatigue, poor concentration, difficulties in communication, and productivity losses from working places. The worldwide imposition of quarantine measures by governments has confined the people to their homes. This global quarantine has not only decreased the use of private and public transportation but has also led to a significant dropdown in commercial activities (3). All these changes have caused a considerable drop in the noise level in most cities in the world. The reports show that noise reductions have gone deep, with seismologists reporting less seismic noise. For example, in Brussels, the seismic noise caused by anthropogenic activities is reported to be down by 1/3 compared with the prelockdown levels (30, 31). Likewise, the decrease in the use of public and private transport along with other commercial activities has caused a significant fall in the levels of noise pollution. With cruises temporarily being on hold, oceans are more in a state of calm. This calmness and decrease in ocean noise is likely to reduce the stress of aquatic creatures. Though the current reduction seems to be a short-term phenomenon, proper and a long-term strategy is needed to check and maintain the environmental noise level within the WHO's permissible limits. As commented by Eulalia Paris, a noise expert and a leading author at EEA, transport sources and other commercial activities are the main causes of noise pollution. As a result, a significant reduction in noise pollution can only be achieved by a long-term and sustainable strategy on the mobility and transportation systems (32).

### Immaculate Beaches

In coastal areas, beaches function as important natural capital assets (2, 3, 5) and provide essential services such as tourism, recreation, sand, land, and source of livelihood to coastal communities (33). Besides providing valuable and intrinsic values, the sandy beaches and dunes are sentinel, shielding the heavy impacts of waves and preventing the furious winds from destroying crops, homes, and other livestock. However, the non-responsible and improper use by people has caused many of the global beaches to present pollution problems (33). These aggregated anthropogenic pollutant impacts are now destabilizing and damaging the potential ability of the beaches and other marine environments to provide key ecosystem services such as coastal livelihood and economic stability, global climate stability, and biological integrity (33). With the global states undergoing lockdown and the WHO declaring emergency and social distancing measures to combat the novel coronavirus pandemic, tourism around the world beaches has been affected. Moreover, the complete closure of various industrial activities has almost halted the pollution from these sources. All these unintended measures have caused a remarkable change in the appearance of many beaches in the world. Prominent examples are the beaches of Salinas (Ecuador), Barcelona (Spain), and Acapulco (Mexico), all these beaches now look cleaner and with clear waters (2). Similarly, Mandal et al. (30) and Saadat et al. (34) while studying the effect of Covid-19 lockdown on the surface water quality, found that the water quality of Vembanad Lake, Kerala, increased significantly. The authors also in their study noticed a significant decrease (34%) in the suspended particulate matter (SPM) concentration of the lake water during the lockdown period. All these studies suggest that the virus crisis has brought with it the unintended benefits for the environment and mankind (16).

### Animals on Street

The environmental changes brought by the coronavirus were first visible from space. Then, as the disease and the lockdown spread, they could be sensed in the sky above our heads, the air in our lungs, and even the ground below our feet. While humans are restricted to their homes under global lockdown, the wild animals all over the planet seem to have come to reclaim their territory. The media outlets are tweeting and uploading several images and videos showing animals on the streets (3). The emergence of wild animals in urban areas is mostly because there is peace and calm, which attracts these animals to the residential areas (accessible at https://climaterealityproject.org/blog/air-pollution-and-coronavirus-connection-explained).

### Feathers Flock Together

While the home confinement rules/lockdown and social distancing have stopped the movement of peoples outside, at the same time, this global lockdown has allowed birds and wildlife to flourish and enjoy all the freedom of nature. Reports confirm that a growing flock of thousands of flamingos beating their black and pink-lined wings has been seen splashing over the glistening water of Nartan Lagoon, of the Adriatic coast. According to park authorities, since January 2020, the number of these birds has been found to increase by 3-fold up to some 3,000. Similarly, the wildlife seems to have regained all their absolute rights and is enjoying the freedom of nature (Agence France–Presse). Similar cases were found in the Indian beaches with flocks of flamingos flying to these beaches with the number increasing by more than 25% compared with previous years.

## Negative Effects of Covid-19 on the Environment

### Covid-19 and the Global Economy

Although the territorial colonization ended long ago, this existing global health crisis can serve as a reminder that the colonization of economics, medicine, and politics are still alive. In addition to its immediate effects on the lives and health outcomes, it is now clear that the coronavirus outbreak is likely to have long-lasting effects on the global economy (35, 36). Loss of lives by any sort of pandemic causes irretrievable damage to the society; however, the Covid-19 pandemic apart from taking a huge toll on the global lives has severely demobilized the global economy. To limit further transmission, governments at local, regional, national, and global levels have decided to undergo complete lockdown. Owing to the complete lockdown and cross-border closure, all the flights, railway services, trucks, buses, and all other types of vehicular transports are suspended. Nearly all the Covid-19-traumatized nations, industries, and entire commercial, educational, religious, and sports institutions are closed. All these restrictions are negatively affecting global economies. Moreover, increased prices, lost income, and overburdened social safety nets will further push the more vulnerable groups into poverty and increase the financial barriers (37). With the production level gone down, the economy of many so-called powerful countries is facing the threat of high inflation. Especially, the gross domestic product (GDP) projections have already been revised downwards in most of the developed countries amid the disruption in production. Most business sectors especially those in tourism, aviation, and hospitality industries are facing serious challenges with a real threat of significant declines in insolvencies, revenue, and job losses (38, 39).

### Effect of Covid-19 on Energy Resources

In the global energy systems, coal stands one of the major fuels accounting for up to 40% of the electricity generation (https://www.iea.org/reports/coal-2019). The global coal production was estimated to have increased by 2.7% in 2018 with the annual production of 8.1 billion tons in 2019. The increase was mainly driven by three major coal-producing countries such as China, India, and Australia, which together accounts for 70% of global production. Owing to the coronavirus lockdown, the global output is expected to increase by 0.5% in 2020. However, due to continuing lockdown and other government policies during the on-going Covid-19 pandemic, the global coal market is likely to fall from $816.5 billion in 2019 to $722.8 billion in 2020. This significant decline in the global output is mainly because of the economic slowdown across the countries caused by the global lockdown to stem the spreading of the Covid-19 pandemic. Similarly, the global oil demand was strongly hit, showing a decline (5%) in the first quarter of 2020. This drastic reduction was mainly because of the curtailments in mobility and aviation which alone accounts for more than 60% of the global oil demand. Likewise, the electricity demand has also shown a significant reduction (>20%) due to lockdown measures, with knock-on effects on the power mix.

### Impacts on Biodiversity

Although affecting all the sectors of human life, the Covid-19 pandemic propagates exponentially and impacts other global resources at an accelerating pace. This global pandemic has its root deep on how we interact, perceive, manage, and conserve global biodiversity. Reports suggest that there is reduced human pressure on natural ecosystems and wildlife (29). The protected areas have witnessed a significant decline in the number of visitors, caused mainly by the travel ban and park closure, reducing the stress on the wildlife. Besides some of the positive effects (all though temporary), it is quite unclear how the conservation biology will fare in the pandemic aftermath. At present, most of the protected areas appear to be safe, and, biodiversity seems to be benefitting from the reduced human activities; however, threats persist especially in the areas where the enforcement has weakened. Although greenhouse gas emissions, environmental pollution, and many other anthropogenic impacts on the wild nature will ricochet, the support and funding for conservation purposes have to compete with a wide range of priorities for financial resources. The forest sector without any doubt is the main contributor to the development of society and for social and economic recovery in the aftermath of any crisis (29). Forests by-products function as essential sources and support the livelihood during the crisis, by delivering necessary products, such as hygiene and sanitary items, respirator papers, ethanol for sanitizer, biomass for heating, and papers for parcel packaging. The negative effects of the Covid-19 pandemic on production and trade of forest and forest by-products will put many of the key livelihoods and industrial sectors at risk (29, 39). Moreover, the forest sector has high rural to urban migration; however, the Covid-19 pandemic is leading to reverse migration, which has the potential to spread the disease to the remote, distant, and unprepared areas. Furthermore, the effect of this global pandemic on forest-based industries will have instant consequences for forest owners and traders arising primarily from the persistent decline in product runoff and sales (European Family Forestry—sustainability in action) (40).

### Other Effects of Covid-19 on the Environment

Since the dawn of civilization, human beings have gradually started manipulating nature for their benefit. Secondly, to satisfy the demands for the ever-growing population, urbanization, and industrialization became quite inevitable and the obvious significance was proved to be detrimental to the global environment (41). Since the outbreak of this novel viral pneumonia, changes to daily life have been swift and unprecedented. As the cases surge and the death toll escalates, both the humans and the environment suffer a lot. Besides the abovementioned ill effects of the Covid-19 outbreak, water bodies and natural and built environments have also experienced significant impacts. For example, to prevent the transmission of coronavirus through wastewater, China has directed the wastewater treatment plants to strengthen the disinfection routines. In contrast, the excessive use of chlorine to treat the water could generate harmful effects on human health (42). Anecdotal evidence indicates that quarantine policies have increased the demands for home delivery, thereby increasing the organic waste production generated by households. Similarly, the increased consumption of medical stuff such as diagnostic supplies, disinfectants, ventilators, N95, and PPE kits, has significantly increased putting the medical waste on the rise; for example, during the coronavirus outbreak, the hospitals in Wuhan China were found to generate an average of 240 metric tons of medical waste per day compared with their previous average of fewer than 50 tons (42). Similarly, in the USA, an increase in garbage production from personal protective equipment has been recorded. The problem got worse, after many countries particularly the USA and the European nations have stopped waste recycling programs in some of their cities, concerning the risk of Covid-19 spreading in the recycling centers (3). Lastly, the impacts of this pandemic on the behavior and psychological well-being are evident. It is well-known that the calamities and other disasters, particularly the ones related to infectious diseases often elicit the waves of heightened fear and anxiety, thus causing massive disruptions to the behavior and psychological well-being of the people. The same is being seen with this virulent creature. Recent studies have shown that the people whether susceptible or not, are developing severe psychological conditions including depression (50.7%), anxiety (44.7%), and insomnia [sleeping disorder (36.1%)] due to lockdown (39, 41).

### Covid-19 Pandemic and the Mental Health

Deadly and disruptive as it already is, the terrible and rapid propagation of Covid-19 pandemic has already induced a considerable degree of concern, worry, and fear among the population in general and certain groups such as care providers, older adults, and people with underlying health conditions in particular. With uncertain prognosis, looming scarcity of medical resources, growing financial losses, the imposition of unfamiliar public health measures that infringe on personal freedoms, and conflicting messages on social media are the major stressors that certainly will contribute to the widespread emotional distress and psychiatric illnesses (43, 44). As mentioned, the public health emergencies have negative effects on the safety, health, and well-being of both individuals and communities. The possible effects on the individuals include stigma, insecurity, confusion, and emotional isolation, while those on the community level include inadequate medical responses due to resource shortage, economic loss, and deficient distribution of necessities, work, and closure of educational institutes (44). All these effects may lead to a range of emotional reactions, unhealthy behaviors, and non-compliance with the public health directives in the population. This is interesting to mention that the previous SARS-CoV-1 epidemics had shown psychiatric symptoms, months after the epidemic was controlled. These indications suggest the possible mental symptoms after SARS-CoV-2 are expected (41). Although the current evidence regarding the direct effect of the Covid-19 pandemic on mental health is scarce, few studies, however, have been carried out indicating that the pandemic has a direct effect on mental health. These authors, while studying the effect of a pandemic on mental health confirmed that the same is affected in the post-pandemic era (43–45). In the population, healthcare workers are regarded as a highly exposed group with a much higher risk of psychiatric symptoms. Although, the number of risk factors of mental health reported is already well-known. Examples of such mental risk factors include present or past medical history, poor self-related health, and female gender. However, the Covid-19 pandemic has added the aspects of self-isolation and quarantine, an established risk factor with psychological impact (46, 47).

## Conclusion

Like the previous catastrophes on the planet Earth, the humans will win over this pandemic in due course of time; however, people should know the limits to which they can thrust nature before it is too late. Environmental changes are arguably the most vital and severe challenge of the twenty-first century. Despite the continuous efforts by governmental and non-governmental organizations to restore and repair nature, humans can only move a few steps forward and yet there are enormous challenges. However, being a blessing in disguise, the Covid-19 pandemic during the past few months has successfully recovered the environment to a much larger extent and has improved the mutually effective link between nature and humans. While at the same time the lockdown and social distancing have contributed positively toward the environment, though, it is essential to take into account the negative effects such as mortality, impacts on social aspects, and the dramatic economic effects as well. The viral pandemic has produced both positive and negative indirect effects on the environment. At present, it is important to control the disease, reduce the transmission, and proactively save lives. Although the positive impacts on the environment may be temporary, the governmental, non-governmental organizations, and the individuals should learn from this lockdown on how to reduce and minimize the pollution on a long-term basis.

## Author Contributions

All authors listed have made a substantial, direct and intellectual contribution to the work, and approved it for publication.

## Conflict of Interest

The authors declare that the research was conducted in the absence of any commercial or financial relationships that could be construed as a potential conflict of interest.
